# Coconuts and Health: Different Chain Lengths of Saturated Fats Require Different Consideration

**DOI:** 10.3390/jcdd7040059

**Published:** 2020-12-17

**Authors:** Susan Hewlings

**Affiliations:** 1 Department of Nutrition and Dietetics, Central Michigan University, Mount Pleasant, MI 48859, USA; hewli1sj@cmich.edu; 2 GRAS Associates/Nutrasource, Guelph, ON N1G 0B, Canada

**Keywords:** saturated fats, coconut, medium-chain fatty acids, lauric acid, cardiovascular disease

## Abstract

The diet heart hypothesis has driven nutrition recommendations and policy for decades. Recent studies have questioned the hypothesis and sparked great controversy over the assumed connection between saturated fat intake and heart disease. Recent evidence suggests that dietary patterns should be the focus of dietary recommendations, not any one food or nutrient. Furthermore, to classify foods as simply saturated fat, polyunsaturated or monounsaturated fats is to ignore the many other potential nutrients and health benefits. Coconut is classified as a saturated fat and therefore listed as a food to limit to reduce heart disease risk. However, different saturated fats, medium-chain or long-chain, act differently metabolically and thus have different health effects. The medium-chain fatty acids predominate in coconut are absorbed differently and have been associated with several health benefits, including improvements in cognitive function and a more favorable lipid profile compared to longer chain fatty acids. Coconuts provide a healthful source of saturated fats and should not be considered the same as foods with longer chain saturated fats. Future recommendations should take this research into consideration. It is the purpose of this review to discuss the research regarding the connection between saturated fat intake, specifically coconut consumption, and health, while focusing on dietary patterns and lifestyle behaviors.

## 1. Introduction

There is a long-standing dogma in public health nutrition that elevated serum total cholesterol, particularly of low-density lipoproteins (LDL-C), increases risk of cardiovascular disease (CVD)/coronary heart disease (CHD). It is also believed that higher high-density lipoprotein cholesterol (HDL-C) decreases risk. Lowering LDL-C has, thus, been at the center of recommendations for reducing cardiovascular disease risk. Connected to this is a long-standing deduction that increased intake of saturated fat increases risk of CVD, and therefore, recommendations to decrease saturated fat intake have also been a part of recommendations for decreasing CVD risk [1,2]. Much of the research linking saturated fat intake to higher LDL-C levels to cardiovascular disease risk has assumed this relationship as part of what is referred to as the diet heart hypothesis [3]. However, the effect of different fats on LDL cholesterol needs to be interpreted cautiously. Without considering CVD risk outcome within the context of the dietary pattern, it is difficult to draw conclusions exclusively from effects on blood lipid levels, especially following short term dietary interventions. Furthermore, it has been shown that in most individuals, reducing saturated fat in the diet reduces large LDL particles subspecies rather than the smaller LDL particles [4]. The smaller particles demonstrate a higher association with CVD risk than do the larger LDL particles [5].

Nonetheless, because of this suggested association, the American Heart Association (AHA) and other major organizations have recommended that individuals decrease their saturated fat intake to reduce their risk of CVD [6,7,8]. However, the saturated fat intake and CVD connection is one of the most debated modern day nutrition beliefs [9,10,11,12,13]. Some studies have shown that this assumption of a direct causal relationship is not completely correct. Lowering saturated fat intake does not always lead to an improved lipid profile, especially if the saturated fat is replaced with carbohydrates [11,12,14]. This points to the importance of considering the replacement fat, as well as the comparator fat or fat source, when interpreting studies related to fatty acids and disease outcomes [15]. Amid this controversy, the AHA and other organizations maintain their overall recommendations to lower saturated fat intake while further clarifying that what the saturated fats are replaced with is important, and that replacement should be with vegetable based polyunsaturated and monounsaturated fatty acids. The AHA has gone on to clarify that different types of polyunsaturated fats, omega 6 and omega 3, as well as different types of saturated fats may have different effects on cholesterol [6]. Within this discussion the consumption of coconut oil is addressed and the AHA’s recommends that individuals avoid consumption of coconut oil. However, the discussion omits research related to the potential benefits of the other nutrients provided by coconut, as well as discussion of the unique properties of lauric acid, the type of saturated fat provided by coconut compared to other saturated fatty acids (SFAs) [16]. Evidence from large observational studies suggests that different SFAs with different physical, chemical, and metabolic structures have different metabolic and health effects, particularly related to blood lipids, glucose-insulin homeostasis, insulin resistance, and diabetes [17,18].

The controversy has evolved from the considerable debate over the last few decades surrounding total fat, particularly saturated fat, and its role in weight management and disease recommendations. Much of the debate has focused on how much saturated fat should be part of a healthy diet [19]. A separate but related controversy is the recent evidence challenging the connection of HDL-C and CVD. While beyond the scope of this review to discuss in detail, the debate centers on whether HDL-C should be a target for intervention and whether recommendations should be centered on a specific target of HDL-C. However, it is agreed that the concentration of HDL-C is an independent, inverse predictor of the risk of having a cardiovascular event; therefore, research should focus on more robust biomarkers and genetic differences [20]. Despite this ongoing debate, it is important to recognize that dietary fats are essential nutrients that play an important role in critical physiological functions including storing energy, supplying essential fatty acids, absorbing and transporting fat-soluble vitamins, protecting and insulating vital organs, providing flavor and satiety in food, providing for cell membrane structure, and serving as a precursor to steroid hormones. However, an abundance of fat in the diet is associated with increased body weight for many reasons, including its caloric density, providing approximately 9 kcal/g compared to approximately 4 kcal/g provided by carbohydrates and proteins [21]. Furthermore, many studies link diets high in fats, especially saturated fats, to many lifestyle diseases such as obesity, heart disease, and cancer [1,22,23]. What has not been clearly identified in the literature and in the recommendations is the food source of the saturated fat and that not all foods classified as saturated fats contain the same fatty acid profiles.

The coconut palm (*Cocos nucifera*) is common across the tropics and subtropics, and while it has become a symbol for warm, sunny vacation locations, it is an important crop for many communities globally, particularly the many islands of the Pacific. It is not just a food source; it provides fiber, timber, fuel, and medicine. It was first used worldwide as an oil when Europeans arrived in the Pacific Islands in the 19th century when it became the first vegetable oil used in world trade [24]. The pharmacological effects of the coconut vary depending on which part of the plant or fruit is being studied or which form consumed (oil, milk, flesh). Production methods (detailed by Krishna et al., 2010 [25]) for extracting coconut oil can vary as well. However, the wet processing method used to extract virgin coconut oil (VCO) is thought to be superior and VCO is thought to have superior health benefits because of its greater phenolic compounds and associated antioxidant properties, while fatty acid profiles seem unaffected by processing [25,26,27]. A recent systematic review reported that antioxidant activity has been identified in the endocarp and coconut water, while the fiber was shown to have antibacterial, antiparasitic, and anti-inflammatory effects [28]. Because of these many bioactive compounds and reported benefits, coconut’s use in a variety of products has gained popularity. It is used in skin care products, cooking oils, sweeteners, beverages, and more. The coconut crop is said to provide sustainable agriculture supporting the three pillars of society, economy, and environment [29]. Despite the many benefits associated with coconut, recommendations for an increase in consumption remain controversial. This controversial recommendation is tied to the debate surrounding saturated fat recommendations and the high saturated fat content of coconut oil, which is about 92%, of which 62–70% is medium-chain triglyceride (MCT) [25,30,31].

The challenge in addressing the issue of whether consumption of coconut, or any other single food, unfavorably effects lipid profiles or cardiovascular disease risk is that food sources typically contain more than one nutrient and diets contain many foods. Therefore, discovering a relationship between any one food and disease must take into consideration the complex nature of dietary and lifestyle patterns that ultimately influence disease outcomes [32].

It is the purpose of this review to discuss the research regarding the connection between saturated fat intake, specifically coconut consumption, and health while understanding that the connection undoubtedly involves more than one food and is more likely reflective of dietary patterns and lifestyle behaviors. However, this is not to say there is no value in exploring the health effects of any given food in the context of a dietary pattern. PubMed and Google Scholar were searched for the following terms: “coconut”, “coconut and health”, “*Cocos nucifera* and health”, “coconut and cardiovascular disease”, “coconut and Alzheimer’s disease”, “coconut antimicrobial”, “coconut and antiviral”, and “lauric acid and health”.

## 2. Coconut Oil

In assessing the relationship between a single food or dietary pattern, it is important to consider the complete nutrient composition of the food(s) being analyzed, even when considering the different forms of the same food [33]. Coconut oil is consumed in three main forms: refined coconut oil, copra oil, and VCO. While the fatty acid profile is the same among the different types, VCO contains a higher amount of monoglycerides and antioxidants and other phytonutrients because of differences in processing [26,34].

**Figure 1:**
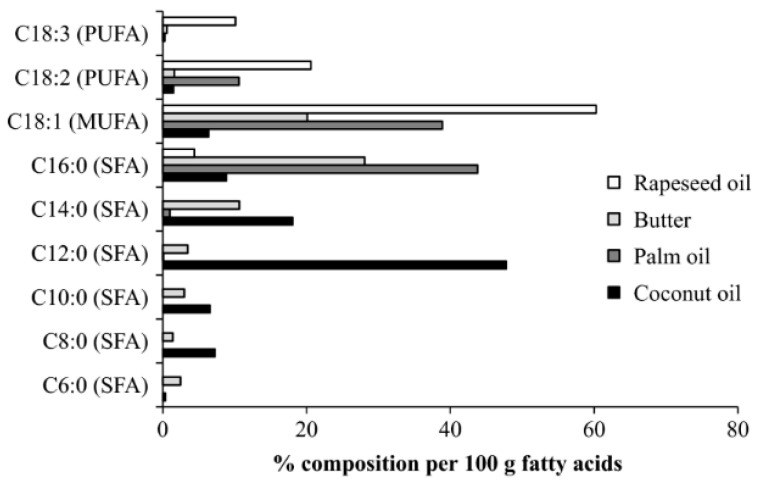
Fatty acid composition of virgin coconut oil compared to other food sources [35]. (With permission from John Wiley and Sons INC., Nutrition Bulletin.)

Saturated fatty acids are not a single family of fats but comprise three sub-groups: short- (C2–C6), medium- (C6–C12), and long-chain (C14–C24) fatty acids. Although all three sub-groups are currently considered saturated fatty acids and are, thus, grouped together in interpreting diet and disease relationships and in making diet recommendations for health, they are distinctly different. Coconut oil is made up of about 92% saturated fats and 9% unsaturated fats. However, these saturated fats differ from the saturated fats in animal fats. The fats in coconut oil are 65% medium-chain and predominantly (~48%) lauric acid (C12) (see Figure 1) [33].

As Figure 1 demonstrates, though butter and coconut oil are both classified as saturated fat, they have very different fatty acid profiles. Coconut oil is the highest natural source of lauric acid. Lauric acid and its derivative monolaurin constitute around 50% of coconut fat-derived lipids [36]. The shorter chain length influences absorption such that medium-chain fatty acids are mostly absorbed intact and transferred via the portal vein directly to the liver on a mixed diet. Medium-chain fatty acids (MCFA) are partly metabolized in the mitochondria of the liver to produce ketone bodies, including 3-β-hydroxybutyrate, acetoacetic acid, and acetone, which are then transported to the organs of the body such as the brain, which can use ketones for energy production [30]. Due to the high concentration of lauric, capric, and caprylic acids, it has been suggested that coconut oil has unique properties different from other foods high in SFAs [37]. It should be noted that the majority of research on MCFAs has been focused on C8:0 and C10:0 fatty acids, although C6:0 to C12:0 are also considered MCFAs, based on their similar properties [33,38]. Because of their absorption characteristics, MCTs have been used since the 1960s in clinical formulas for adult and infant patients who have issues with the absorption of longer chain fats. Because C12:0 diffuses into the mitochondrial membrane without requiring the carrier carnitine, unlike longer chain fatty acids, it has historically been used for sports nutrition products when quicker absorption and utilization are desirable [39]. Lauric acid can be classified as either a long-chain or MCFA, depending on digestion and absorption and what else is being consumed in the diet. For example, it has been reported that when subjects consume a diet containing different fats, MCFAs tend to be channeled towards the portal vein. However, when the diet is mainly MCFA, some of these may also be incorporated into chylomicrons, suggesting the body adjusts based on the fat content of the diet [40]. This may explain some of the conflicting results that are reported in the literature [33]. Because of these differences in properties and its health benefits, lauric acid should not be considered the same as other longer-chain saturated fatty acids, such as palmitic acid (C16:0) and stearic acid (C18:0), which are much more prominent in butter and animal fats. For example, it has been reported that coconut oil consumption leads to a more favorable lipid profile compared to consumption of butter [38].

## 3. Cardiovascular Risk Factors

### 3.1. Saturated Fat and CVD/CHD

There is debate surrounding the role of SFA in the development of CVD, and since coconut oil is largely saturated fat (92%), its consumption has also been discouraged due to “guilt by association”. Several studies suggest no relationship between saturated fat consumption and CVD. For example, a meta-analysis of prospective cohort studies found no significant evidence that dietary saturated fat is associated with an increased risk of cardiovascular disease [11]. However, the study had limitations in that it compared diets high in saturated fat to commonly eaten diets high in carbohydrates, specifically high in refined carbohydrate and added sugar, and failed to distinguish between the type of carbohydrate being substituted. Consistent with these findings, a prospective cohort study (the PURE study) conducted over 10 years of 135,335 individuals assessing dietary intake with validated food frequency questionnaires from 18 countries, on five continents, where overnutrition is common in some and undernutrition in others, found that high carbohydrate intake (>60% total calories) was associated with increased risk of total mortality. In addition, total fat as well as saturated fat and unsaturated fat intake, which were considered separately, were associated with lower mortality rate [41]. The authors suggest that their findings contradict those reporting a connection between saturated fat and CVD [22] because many of these studies were conducted in homogenous populations where CVD is prevalent and saturated fat intake is higher than in many lower income countries, making applicability to diverse populations challenging. It should be noted that many participants in the PURE study consumed as high as 77% of their daily calories from carbohydrates, which could explain some of the findings related to carbohydrate intake and mortality. They did not differentiate types of carbohydrates either, which limits interpretation [41]. Similarly, a meta-analysis of observational cohort studies (3–12 depending on the association) reported no association between higher intake of SFA and all-cause mortality, CHD, CHD mortality, ischemic stroke, or type 2 diabetes among healthy adults [14]. A meta-analysis of 21 prospective epidemiological studies ranging from 6–21 years in length using various dietary assessment tools reported no association between SFA intake and CVD or stroke, even when controlling for energy intake [11]. In contrast, were the findings of a prospective cohort study followed 84,628 women (Nurses’ Health Study, 1980 to 2010), and 42,908 men (Health Professionals Follow-up Study, 1986 to 2010) who were free of diabetes, cardiovascular disease, and cancer at baseline. Diet was assessed by semiquantitative food frequency questionnaire every 4 years, and saturated fat intake, unsaturated fat intake, and sources of carbohydrates were compared in relation to risk of CVD. They documented 7667 incident cases of CHD and found that replacing SFA with monounsaturated fatty acids (MUFAs), polyunsaturated fatty acids (PUFAs), or carbohydrate from whole grain is associated with lower CVD risk [23]. Furthermore, when reviewing studies using a multivariable regression analysis that isolates the effects of specific nutrient exchanges, replacing 5% energy intake from SFAs with equivalent energy intake from MUFAs, PUFAs, or whole grain sources of carbohydrate was associated with a significantly lower risk of CHD [16]. The results of these studies strongly support careful examination of the balance of nutrients such that with what one replaces the decreased nutrient is an important aspect of assessing study outcomes, in this case with what one replaces saturated fat appears to influence disease risk. This is supported by results from two large prospective cohort studies (the Health Professionals Follow up and the Nurses’ Health Study), as neither found significant associations between saturated fatty acid intake and risk of cardiovascular disease when they controlled for the replacement nutrients [22,23,42]. In addition, the source of the SFA should be considered. A report from the Rotterdam prospective cohort study of 4722 Dutch men and women over 55 years of age found that higher intake of palmitic acid was associated with a higher risk of CHD, while SFAs with other carbon chain lengths were not. Substitution of total SFA with animal protein was also associated with CHD, while no association was found between total SFA intake and CHD risk regardless of food source [43]. Similarly, results from the Atherosclerosis Risk in Communities (ARIC) study suggest that consumption of plant-based protein and fats were associated with lower mortality, while proteins and fats from animal sources were associated with increased mortality, suggesting that the sources of food and thus SFA chain length may impact disease risk [44]. In a 10.6 years prospective cohort study of 195,658 participants from the UK Biobank, there was no evidence that saturated fat intake was associated with incident CVD, while substitution of polyunsaturated for saturated fat was associated with higher CVD risk. They also reported a positive correlation between saturated fat intake and all-cause mortality at intakes above average consumption. Interestingly, results from this study suggest that the dietary intake with the lowest hazard ratio for all-cause mortality consisted of a high intake of fiber (10 to 30 g/day), protein (14% to 30%), and monounsaturated fat (10% to 25%) and moderate intake of polyunsaturated fat (5% to <7%) and starch (20% to <30%) [45].

### 3.2. Type of Fat

The information from the Rotterdam and ARIC studies suggests that in addition to considering what the SFA is replaced, it is important to consider the type of saturated fats as well. It has been suggested that lauric acid and coconut oil do have an impact on lipoproteins. A systematic review and regression analysis published by the World Health Organization of SFAs on lipid profile and lipoproteins found that reducing SFA intake with cis-PUFA (of mostly linoleic acid or α-linolenic acid) or cis-MUFA (predominantly oleic acid) were more favorable than replacing SFA with a mixture of carbohydrates. Cis-PUFA replacement had more favorable effects on total and LDL cholesterol and triglycerides. The effects were seen even at low saturated fat intake. Increased intake of lauric, myristic, or palmitic acid raised serum total, LDL, and HDL cholesterol levels, and lowered triglyceride levels more than complex carbohydrate replacement, while increased intake of stearic acid did not have a significant effect. Lauric acid decreased the total cholesterol to HDL cholesterol and LDL cholesterol to HDL cholesterol ratios compared to a mixture of carbohydrates. The studies included in this analysis utilized tightly controlled diet interventions were short term (3–5-weeks), and included a range of SFA intakes, from 1.6–24.4%. While this allows for precise detection of effects, it may not reflect real world changes in diet, which typically involve more than one nutrient. Furthermore, long-term effects are not reflected in the short-term studies. The authors point out that intakes of lauric and myristic acid in the included studies was low (i.e., mean of 1.2% of total energy intake). They suggest that “in order to obtain more insight into the effects of these two SFA on the serum lipoprotein profile at higher intakes, more well-controlled intervention studies at higher intakes are needed” [46]. In a systematic review and meta-analysis, coconut oil was found to significantly increase HDL-C when compared with plant oils and animal oils and it significantly increased LDL-C compared with plants oils and lowered LDL-C compared with animal oils [37]. Similarly, two studies which used VCO as the coconut source reported significantly elevated HDL-C and no significant changes in LDL-C when comparing subjects consuming VCO to those consuming a virgin olive oil/butter/standard diet [47,48]. In another study, coconut oil was found to be positively associated with HDL-C levels in menopausal women in the Philippines [49].

In a randomized trial, participants were randomized to 1 of 3 groups, extra VCO, virgin olive oil, or butter and asked to add 50 g of one of these fats per day for four weeks into their normal diet or consume it as a supplement. This study found that LDL-C concentrations were significantly increased in the butter group compared with the extra VCO or olive oil group. No differences in LDL-C were observed in coconut oil compared with olive oil. Coconut oil also significantly increased HDL-C compared with butter or olive oil [47]. Similarly, in a study comparing coconut oil, butter, and safflower oil on lipids and lipoproteins in free-living subjects with moderately elevated cholesterol levels, it was found that the coconut oil and butter diets significantly increased total cholesterol and LDL-C relative to safflower oil; however, total cholesterol and LDL-C were significantly higher on the diet containing butter than on the diet containing coconut oil. The authors concluded that “coconut oil rich in lauric acid has a lesser effect than butter, which is high in palmitic acid, on total and LDL cholesterol in hypercholesterolemic men and women” [50].

A recent systematic review and meta-analysis assessing the effect of coconut oil consumption on blood lipids and other cardiovascular risk factors compared with other cooking oils included 16 articles published through June 2019. Coconut oil consumption significantly increased LDL-C by 10.47 mg/dL and HDL-C by 4.00 mg/dL as compared with non-tropical vegetable oils [51]. It should be noted that the authors did not compare coconut consumption to animal sources of saturated fat and they included shorter term studies with no evidence of cardiovascular outcomes.

A previous review conducted a network meta-analysis, which may be more appropriate for comparing multiple treatments, in order to compare the effects of 13 different oils (safflower, sunflower, rapeseed, hempseed, flaxseed, corn, olive, soybean, palm, coconut oil, beef fat, lard, and butter) across randomized trials on established blood lipid factors. Fifty-four trials with 2065 participants published between 1984 and 2018 were included in the analysis. Coconut oil was ranked best to improve HDL-C. The analysis showed that all vegetable oils including coconut oil were more effective in reducing total cholesterol (−0.49 to −0.18 mmol/L) and LDL-C (−0.42 to −0.23 mmol/L) compared with butter [52].

A systematic review was conducted to assess the effect of coconut consumption on cardiovascular risk factors and outcomes in humans. The review included 8 clinical trials and 13 observational studies examining the effects of coconut oil or coconut products on serum lipids. The authors reported that in most studies, coconut oil raised total and LDL-C to a greater extent than cis unsaturated plant oils, but to a lesser extent than butter, suggesting that while coconut oil consumption may offer some benefits compared to other saturated fats, they most likely do not offer a benefit compared to other vegetable fats. However, this is not to say that consumption of coconut unfavorably raises serum lipids. For example, the observational studies included in the review suggested that consumption of coconut flesh or squeezed coconut in the context of traditional dietary patterns does not lead to adverse cardiovascular outcomes. This is an important observation, as it addresses the modern view of the effects of dietary intake on disease outcomes from the perspective of dietary patterns as opposed to one particular food or nutrient. Thus, considering all nutrients, phytochemicals, and other bioactive substances provided by foods that make up dietary patterns [38]. This is an important step forward to providing more appropriate guidelines and recommendations

To summarize, evidence suggests that the saturated fat lipid CVD risk connection is being appropriately challenged, please see a summary of included studies in Table 1. As discussed above, it appears that the connection is not as linear, nor as simple as previously suggested in several ways. It is not as simple as just decrease saturated fat and then disease risk decreases, it appears that with what one replaces the saturated fat is of great importance, in that replacing saturated fat with carbohydrates does not seem to be as advantageous as replacing it with vegetable sources of mono and poly unsaturated fats. Additionally, recent research has shown that not all saturated fats are the same, and therefore, do not have the same effect on CVD risk. Shorter chain saturated fats from vegetable sources appear to have a more beneficial effect on blood lipid profiles and potentially any associated CVD/CHD risk than do longer chains. Coconuts are classified as a saturated fat, although the majority of fats contained in the coconut are medium-chain, and therefore, appear to have a more favorable impact on blood lipids and CVD/CHD risk than animal based saturated fats that are of a longer chain such as beef fat and butter. Oils contain more than just the fatty acids that comprise them, and other nutrients, such as polyphenols and vitamins, may also have an impact on aspects of health in addition to their impact on CVD risk. More research is needed in order to accurately differentiate the types of saturated fats and their impact on health outcomes. Within such research, it would be important to consider all bioactive ingredients within the various sources such that all beneficial compounds are considered in addition to the fatty acid content. It is critical that future revisions of public health policy and dietary recommendations consider such evidence.

## 4. Alzheimer’s Disease

While the majority of research and discussion surrounding the effects of coconut consumption on health have focused on cardiovascular health, there is evidence that coconut consumption may positively impact other diseases. Alzheimer’s disease (AD) is a progressive neurogenerative disease and is the most common type of dementia [53]. While an exact cause is not known, lifestyle factors similar to those associated with CVD have been suggested to be associated with an increased risk of AD. Chronic inflammation and increased oxidative stress are thought to be at the center of lifestyle factors that increase AD risk [54]. Therefore, dietary patterns as well as individual foods and nutrients have been investigated for their potential role in the prevention of AD [55]. One such food is coconut. There are a multitude of claims, case studies [56], small clinical trials [57], and anecdotal evidence in the lay literature, but well-controlled clinical trials in humans to support any efficacious claims are lacking [58]. However, a reasonable mechanism of action does seem to support further investigation into the role coconut oil, and the MCTs it contains, may play in improving cognition in those suffering from early stage cognitive dysfunction [30]. In addition to its neuroprotective antioxidant properties, coconut contains high levels of MCFA, which lead to ketone body formation. The ketones are then thought to offset an early sign of AD, brain glucose hypometabolism, by providing an alternative energy source [59,60]. A pilot study was conducted to assess cognitive changes in 44 subjects with AD. Patients (*n* = 22) received a coconut-enriched Mediterranean diet for 21 days and the control (*n* = 22) received an isocaloric Mediterranean diet with no coconut. Those consuming the coconut enriched diet experienced significant improvements in episodic, temporal orientation and semantic memory. The improvements were greater in women with mild-moderate state AD [57]. While there are no large clinical trials or cohort studies to make recommendations for consuming coconut oil to improve cognitive function, information from mechanism of action and small studies supports further research in this area [30,55].

## 5. Antimicrobial/Antiviral

Coconut has a long history of use as both a food and medicine throughout the world. Its use in Ayurvedic medicine was documented 4000 years ago [61]. The MCFAs are said to be beneficial in destroying a wide assortment of lipid-coated bacteria by disintegrating their lipid membrane [62]. Coconut oil has been shown to demonstrate antifungal and antimicrobial properties, specifically against *P. aeruginosa*, *E. coli*, *Proteus vulgaris*, and *Bacillus subtilis* [63,64] and on Vibrio bacteria [65]. Lauric acid, capric acid, and caprylic acid have been shown to inhibit bacterial growth [66,67,68]. It has also been shown to have a significant effect on polymicrobial dental biofilm activity [69]. In viruses they are thought to interfere with signal transduction and interfere with the virus assembly and maturation [62,70]. In summary, the use of VCO for its antimicrobial and antiviral benefits have a long history of use and several studies have supported its use, warranting further studies in humans to further define its antimicrobial and antiviral benefits.

## 6. Conclusions

The coconut palm is an important crop for communities globally both as a food source as well as for fiber, timber, and medicine. It contains several bioactive compounds, particularly extra virgin coconut oil. Many of its health benefits have been overlooked due to its fatty acid composition. It contains primarily saturated fatty acids. The consumption of a diet high in saturated fatty acids has been connected to an increased risk of CVD and other major lifestyle disease. Therefore, historically, public health recommendations have focused on messages encouraging the public to decrease consumption of saturated fats, including coconuts. However, the connection between saturated fat consumption and CVD has been questioned recently. When determining if decreasing saturated fat intake improves lipid profiles and potentially CVD risk it is clear that what the saturated fat is replaced with is critical to interpreting the results. Replacing the fat with vegetable fats appears to be more beneficial than replacing them with simple carbohydrates. This is not surprising considering the negative health implications of consuming excess simple carbohydrates. While diet–disease relationships cannot be definitively identified based on one food or food group, it is clearly important to identify food sources of nutrients when assessing such relationships. For example, there is significant data to support differentiating different food sources of saturated fats to clarify the unique nutrients of each source and the associated health impact. This would be similar to the approach taken with different types of polyunsaturated fats, whereby omega 3 and omega 6 polyunsaturated fats are recognized for their unique properties and health impact. Taking the same approach with different types and sources of saturated fatty acids is warranted when one considers the unique nutrient composition of the coconut compared to other saturated fat sources, such as beef. Beef contains mainly the longer chain saturated fatty acids while coconut contains medium-chain. The medium-chain fatty acids are absorbed differently and have been associated with several health benefits including improvements in cognitive function and a more favorable lipid profile compared to longer chain fatty acids. In the context of a dietary pattern that has been associated with health benefits such as the Mediterranean diet, coconuts may, therefore, provide a healthful source of saturated fats while also providing phenols and antioxidants. While the evidence related to the connection between saturated fat intake and heart disease may be inconclusive and thus not be in full support of reversing suggestion to avoid saturated fats, certainly a revision is justified. A more in-depth investigation of the connection between different types of saturated fat and disease is warranted. Future recommendations and guidelines should take all aspects of the research into consideration when compiling revisions and associated policy.

## Figures and Tables

**Table 1:** Summary of studies related to fats and CVD.

Author	Type of Study	Purpose	Included	Conclusion
[11]	Meta-Analysis of prospective epidemiological studies	21 studies related to the association of dietary saturated fat with CHD, stroke, CVD; CHD inclusive of stroke.	5–23 years of follow-up of 347,747 subjects	No significant evidence that saturated fat is associated with increased risk of CHD or CVD
[41]	Epidemiological Cohort	Associations between consumption of carbohydrate, total fat, and each type of fat with cardiovascular disease and total mortality.	Dietary intake of 135,335 individuals aged 35–70 years, in 18 countries; median follow-up 74 years,	↑ Carbohydrate intake associated with ↑ risk of total mortality; total fat and types of fat related to ↓ total mortality. Total fat and types of fat not associated with CVD, myocardial infarction, or cardiovascular disease mortality, saturated fat had an inverse association with stroke.
[22]	Prospective Cohort	Associations between intakes of individual SFAs and their food sources in relation to the risk of CHD.	80,082 women aged 34–59 Nurse’s Health Study; 14-year follow up	Short- to medium-chain fats not associated with CHD risk; intake of longer chain = ↑ risk; ratio of PUFA to SFA inversely associated with CHD risk.
[14]	Meta-Analysis of observational studies	Associations between intake of saturated fat and trans unsaturated fat and all cause mortality, CVD and associated mortality, CHD and associated mortality, ischemic stroke, and type 2 diabetes.	41 studies on saturated fats and health outcomes in prospective cohort studies published between 1981 and 2014; 67 data points; 20 studies with 28 data points for trans fats	Saturated fats are not associated with all cause mortality, CVD, CHD, ischemic stroke, or type 2 diabetes, but the evidence is heterogeneous with methodological limitations. Trans fats are associated with all cause mortality, total CHD, and CHD mortality.
[23]	Prospective Cohort	Associations of SFAs compared to PUFAs and carbohydrates to CHD risk.	84,628 women 42,908 men 24–30 year follow up	↑ Intake of PUFAs and whole grains = ↓ risk of CHD; replacing 5% of energy intake from SFAs with PUFAs, MUFAs, or carbohydrates from whole grains was associated with a 25%, 15%, and 9% ↓ risk of CHD.
[42]	Prospective Cohort	Association between fat intake and CHD.	43,757 men aged 40 to 75 years; 6 year follow up	SFA intake not associated with CHD once corrected for fiber intake; ↑ linolenic acid = ↓ risk CHD.
[43]	Prospective Cohort	Association between dietary SFA and CHD depends on the food source, the carbon chain length of SFA, and the substituting macronutrient.	4722 Dutch men and women > 55 years; 16.3 year follow up	↑ Intake of palmitic acid ↑ risk; other SFAs are not associated with CHD; no effect of food source of SFA; replacement of SFA with animal protein ↑ risk; replacement with other macronutrients not associated with risk.
[44]	Prospective Cohort and Meta-Analysis	Association between carbohydrate intake and mortality.	15,428 aged 55–64 years ARIC study; 25 year follow up	U-shaped relationship between carbohydrate intake and mortality; low carbohydrate intake with animal protein increased risk, while with high plant protein decreased risk.
[45]	Prospective population study	Association of macronutrient intake with all cause mortality and CVD, and the implications for dietary advice.	195,658 adults 10.6 year follow up	Carbohydrate intake > 50% ↑ association with mortality; ↑ intake of MUFA, ↓ intake of PUFA, ↓ intake of SFA = ↓ risk of mortality.
[46]	Systematic Review and Meta-Analysis of RCTs	Assess the impact of phytosterol (PS) supplementation on serum Lp(a) and FFA concentration.	12 effect sizes from 7 different studies	PS supplementation = ↓ in Lp(a) and FFA.
[37]	Systematic Review and Meta-Analysis	Examine the evidence surrounding coconut oil consumption and its impact on cardiovascular health.	12 studies	Compared with plant oils and animal oils, coconut oil ↑ HDL-C by 0.57 mg/dL and 0.33 mg/dL. Coconut oil significantly ↑ LDL-C by 0.26 mg/dL compared with plant oils and ↓ LDL-C (48.1%) compared with animal oils. No effects on triglyceride. Better lipid profiles were demonstrated with the virgin form of coconut oil.
[49]	Prospective Cohort	Association between coconut oil intake and plasma lipid profiles.	1896 Filipino women aged 35–69 years	In pre-menopausal women, dietary coconut oil use was associated with TC and HDL-C, not in post-menopausal women; coconut oil did not elevate TC, triglyceride levels, and TC/HDL.
[47]	RCT	Compare changes in blood lipid profile, weight, fat distribution, and metabolic markers after four weeks of consumption of 50 g daily of extra virgin coconut oil, butter, or extra virgin olive oil.	91 men and women	LDL-C significantly increased on butter compared with coconut oil and with olive oil, no differences in change of LDL-C in coconut oil compared with olive oil. Coconut oil significantly increased HDL-C compared with butter or olive oil. Butter significantly increased TC/HDL-C ratio and non-HDL-C compared with coconut oil, while coconut oil did not significantly differ from olive oil for TC/HDL-C and non-HDL-C. No significant differences in changes in weight, BMI, central adiposity, fasting blood glucose, and systolic or diastolic blood pressure in any group.
[50]	RCT cross over	Compare the effects of coconut oil, butter, and safflower oil on lipids and lipoproteins of moderately hypercholesterolemic subjects.	13 men and 15 women with a plasma total cholesterol between 5.5 and 7.9 mmol/L and plasma triacylglycerols (TAG) less than 3 mmol/L consumed 50% of fat from butter, coconut oil, or safflower oil	Coconut oil and butter diets increased TC and LDL-C compared to safflower oil; the levels of both were significantly lower in the coconut oil than on the butter diet.
[51]	Systematic Review and Meta-Analysis	A systematic review of the effect of coconut oil consumption on blood lipids and other cardiovascular risk factors compared with other cooking oils using data from clinical trials.	16 articles included in the meta-analysis	Coconut oil increased LDL-cholesterol by 10.47 mg/dL and HDL-cholesterol by 4.00 mg/dL compared with nontropical vegetable oils. Coconut oil consumption did not significantly affect markers of glycemia, inflammation, and adiposity as compared with nontropical vegetable oils.
[52]	Systematic Review and Network meta-Analysis	Compare the effects of different oils/solid fats on blood lipids.	54 RCTs 2065 subjects included	Safflower oil ↓ in TC and LDL-C the most, followed by rapeseed oil and sunflower oil; soybean oil was the most effective oil to ↓ TG, followed by corn oil and palm oil; butter and lard were ranked worst for TC and LDL-C reduction; coconut oil was ranked best to ↑ HDL-C, followed by palm oil and beef fat. The NMA showed that all vegetable oils were more effective in reducing TC and LDL-C compared with butter.
